# Determinants of private health insurance uptake and its association with healthcare utilization in Gulf Cooperation Council countries: a systematic review

**DOI:** 10.1080/16549716.2026.2647528

**Published:** 2026-03-25

**Authors:** Khaled Shaeel Althabaiti, Mohammad Badrul Bhuiyan, Monica Hunsberger, Sayem Ahmed, Jahangir Khan

**Affiliations:** a School of Public Health and Community Medicine, Institute of Medicine, Sahlgrenska Academy, University of Gothenburg, Gothenburg, Sweden; b Basic Nursing Sciences Department, College of Nursing, Taif University, Taif, Saudi Arabia; c Department of International Public Health, Liverpool School of Tropical Medicine, Liverpool, UK; d Health Economics Research Group (HERG), Department of Health Sciences, Brunel University London, Uxbridge, London, UK; e Department of Learning, Informatics, Management and Ethics, Karolinska Institutet, Stockholm, Sweden

**Keywords:** Determinants, Gulf Cooperation Council, private health insurance, healthcare utilization, insurance

## Abstract

All Gulf Cooperation Council (GCC) countries have a multi-payer healthcare system that comprises governmental health coverage (GHC), funded by the government, and private health insurance (PHI), mainly sponsored by employers and purchased by individuals. Both are expected to influence healthcare utilization and contribute to system efficiency and patient well-being. This systematic review explored the determinants of PHI uptake and its association with healthcare service utilization in the presence of GHC in GCC countries. We systematically searched CINAHL, PubMed, Scopus, Web of Science, and Cochrane Library for peer-reviewed studies published between January 2012 and October 2022. Study quality was assessed using the Critical Appraisal Skills Programme (CASP) checklists for both quantitative and qualitative studies, following PRISMA guidelines. Twenty-six studies met the inclusion criteria. Determinants of PHI uptake were mapped to Andersen’s Behavioral Model of Health Services Use (BMHSU) and categorized into (1) predisposing factors (sex, age, marital status, and education), (2) enabling factors (employment/income and health system-related factors such as access and perceived service quality), and (3) need factors (health status, including chronic noncommunicable diseases). PHI uptake was positively associated with being male, married, highly educated, employed with a high income, and having chronic diseases. PHI was positively associated with healthcare utilization, particularly routine check-ups, preventive services, and the use of prescribed medicines. In GCC countries, PHI uptake is influenced by sociodemographic and socioeconomic characteristics, health status, and perceived service quality. PHI is also associated with higher healthcare utilization, underlining the need for evidence-informed policies that enhance equity and expand coverage.

## PAPER CONTEXT

- **Main finding:** Private health insurance uptake across Gulf Cooperation Council countries was influenced by sociodemographic and socioeconomic characteristics, health status, and the quality of services. Additionally, private health insurance was likely to be associated with higher utilization of healthcare services.
- **Added knowledge:** This systematic review identifies how shifts in health policy across Gulf Cooperation Council countries, particularly the move from universal public coverage toward mixed systems combining governmental and mandatory private insurance which have redefined the determinants of private health insurance enrollment and healthcare utilization. It highlights how socioeconomic characteristics and policy reforms interact to shape access patterns under differing insurance schemes.
- **Global health impact:** The findings can support policymakers in developing equitable and sustainable health financing reforms toward universal health coverage.

## Background

The Gulf Cooperation Council (GCC) comprises six countries: Kuwait, Bahrain, the United Arab Emirates (UAE), the Kingdom of Saudi Arabia (KSA), Qatar, and Oman. These countries have similar financial situations, such as dependence on vast reserves of oil and gas [1]. In recent decades, these countries have experienced considerable population growth, partly due to increases in expatriate populations [2]. The percentage of expatriates in these countries ranges between 33.3% in Oman and 86.2% in Qatar [3]. In the past few decades, healthcare investments in GCC countries have increased through the expansion of healthcare facilities, including large medical complexes and medical cities [4]. According to recent estimates in 2019, the GCC member countries spent on average 4.6% of their gross domestic product (GDP) on healthcare expenditure, ranging from as low as 3.5% (Qatar) to as high as 6.1% (KSA) [5].

However, the increasing demand for health services puts a significant burden on the health system in GCC countries, and thus, there is a need for substantial changes in the strategies and infrastructure of the healthcare system to overcome these challenges [6]. In response to financial hardship, governments in GCC countries have implemented reforms to expand health coverage for their populations and improve access to healthcare by adopting private health insurance (PHI) schemes in addition to governmental health coverage (GHC), with the broader aim of achieving universal health coverage (UHC) [7]. Accordingly, it has been reported that there are approximately 214 PHI companies in GCC countries; 90 of these companies have a major market share (80% of the total premium collected), while the other 114 companies compete for the remaining 20% of the market share [8]. The GCC countries have similar policies for governing many aspects of healthcare services and health insurance [9]. It should be noted that all GCC countries have a multipayer health financing system, comprising GHC, which the government fully funds, and PHI, either sponsored by employers or purchased by individuals [10].

As of 2022, non-citizens accounted for 54.6% of the total population across GCC countries, compared with 47.8% in 2000, with their share exceeding 85% in Qatar and the UAE. This reflects a demographic transformation that has steadily intensified over the past two decades, driven by sustained labor migration, and now carries significant implications for healthcare financing and equity [11,12].

Fadhil et al. (2022) argued that extending mandatory PHI to low-income expatriates in GCC countries may enhance health equity. In addition to this equity goal, the policy also aimed to reduce the financial burden on governments by shifting healthcare costs primarily to employers, with a smaller share borne by individuals. Nevertheless, this approach may place additional financial and operational pressure on the healthcare system [6]. This pressure may stem from increased service demand without a proportional investment in infrastructure, especially when some individuals with PHI lack sufficient understanding, which can lead to inappropriate use, including unnecessary visits that strain resources and reduce system efficiency [6,13]. Ram (2014) added that achieving healthcare targets in the GCC countries will depend on responding to new challenges, such as health insurance coverage, insufficient medical practitioners, and a lack of stringent guidelines on quality standards [14]. Similarly, Alshamsan et al. (2017) stated that the path to a universal insurance scheme in the GCC countries would face challenges, such as an increasing number of non-national residents, which highlights the need for inclusive and sustainable policy solutions [9].

Under the GHC in KSA, public health facilities offer free healthcare services to Saudi citizens and expatriates working in various governmental sectors (Ministry of Health and Ministry of Education) [15]. To reduce the financial burden on the government, KSA introduced the Cooperative Health Insurance Law, which mandates employers to provide private insurance coverage for all workers (both Saudi and non-Saudi) in the private sector [16]. This strategy aimed to overcome the challenge of providing free healthcare services to the residents in the country, which has been achieved through 27 insurance companies offering medical coverage to more than 11 million people [17]. The regulations and laws for PHI issued by the Saudi Council of Health Insurance (CHI) outline how healthcare services are financed. Responsibilities are shared between employers (who pay premiums), employees (who make co-payments), beneficiaries (who must understand how to access care), and insurance companies (which manage coverage and claims) [16].

In Kuwait, citizens receive free healthcare services at public health facilities, whereas foreigners are required to pay a compulsory annual health fee to access medical treatment at these facilities. However, the costs of services provided by private healthcare providers are not covered under this requirement. Renewal of a residence permit for expatriates requires payment of the health fee by their employer. Even when individuals have paid the annual health fee, they may still have to pay for certain services provided by public facilities [18].

In Bahrain, the healthcare system depends on both public and private healthcare providers to deliver primary care services. Bahraini nationals are covered under the National Social Health Insurance Program (Sehati), which entitles them to free services at public healthcare facilities funded by the government. Non-Bahraini residents are charged a small fee to receive healthcare services at government facilities; however, Sehati also extends cost-free coverage to specific expatriate groups, such as domestic workers (e.g. drivers and gardeners) sponsored by their employers. Other expatriates working in the private sector must be covered through employer-sponsored PHI [19].

Oman provides free GHC to all Omani citizens through the Ministry of Health. In addition, Oman has introduced a compulsory PHI scheme named ‘Dhamani’, which provides coverage for more than 2 million individuals. The program includes individuals working in the private sector, expatriates, and international visitors. The responsibility of employers is as follows: Dhamani mandates that employers cover the cost of health insurance premiums for their employees. The program ensures essential health coverage, including hospitalization, emergency care, treatment for common illnesses that affect work performance, and medications prescribed by certified doctors. Employers have the option to provide extra benefits such as maternity, infant health, dental, and vision care [20].

Qatar provides publicly funded healthcare to all residents through the Hamad Medical Corporation. Qatari citizens receive free healthcare services under the GHC system. For migrant workers, there are two strategies to improve healthcare access, and all expatriates must be covered by one of these strategies to obtain or renew their residency. The first is the Hamad Health Card, which grants residents, including migrant workers and their families, access to public healthcare facilities. Under this program, migrants pay minimal fees for the medical treatment they receive. The second is employer-sponsored PHI, which may be advantageous for skilled migrants, as it offers PHI plans provided by employers that provide international coverage [21].

In the United Arab Emirates (UAE), citizens receive free healthcare at health facilities under the GHC system, while expatriates are required to have PHI to access healthcare, which is mandatory for obtaining residency. In contrast, citizens can utilize certain private healthcare facilities covered by GHC. The Emirates of Dubai and Abu Dhabi have each established unified GHC programs, ‘Enaya’ for Dubai citizens and the ‘Thiqa’ program for Abu Dhabi citizens, thus providing access to high-quality medical services and better healthcare through an advanced integrated system, according to a press release [22].

Although the GCC countries have similar policies for governing many aspects of healthcare services and insurance, differences in implementation create a valuable opportunity to widen knowledge about the factors associated with the uptake of PHI and its impact on the utilization of health services [9]. Furthermore, due to the recent introduction and adoption of PHI in GCC countries, it is necessary to evaluate its implementation, influencing factors, and effects on healthcare utilization. Such research could help policymakers shape the future of the health system in the GCC countries. Therefore, the current study aims to explore the determinants of PHI uptake in GCC countries and its association with the utilization of health services.

The study aims to answer two specific questions:

1. What are the determinants of PHI uptake in GCC countries?
2. What is the association between PHI and healthcare utilization in GCC countries?

## Methods

Two reviewers searched the following electronic databases and search platforms: CINAHL, PubMed, Scopus, Web of Science, and Cochrane Library to identify relevant English-language studies published between 1 January 2012 and 31 October 2022. To ensure a broad range of relevant studies, we used a combination of health insurance-related Medical Subject Headings (MeSH) terms and text words (ti, ab, kw) to search the databases. Our study primarily focuses on private health insurance (PHI). To ensure comprehensive coverage, we included all MeSH and text terms related to health insurance, which allowed us to capture studies where PHI status was discussed either directly or indirectly (e.g. through out-of-pocket spending or willingness to pay). Although the search strategy included the generic term ‘health insurance,’ we operationalized eligibility as PHI and confirmed the coverage type during full-text screening. Studies were classified as PHI only when the described health insurance was private/employer-based, the typical form of insurance in GCC settings. This interpretation is consistent across the literature, particularly when referring to insurance coverage among expatriates or workers in the private sector, where PHI is the predominant form of insurance. This understanding was carefully verified during the full-text screening stage. The following search strategy was used: (‘insurance, health’[MeSH Terms]) OR (health insurance*[tiab]) OR (‘insured’[Title/Abstract]) OR (‘uninsured’[Title/Abstract]) OR (‘community insurance’[Title/Abstract]) OR (‘medical insurance’[Title/Abstract])) OR (‘universal healthcare’[Title/Abstract])) AND (GCC) OR (Gulf Cooperation Council) OR (Bahrain*) OR (Saudi) OR (KSA) OR (Kuwait*) OR (Oman*) OR (Qatar*) OR (UAE) OR (United Arab Emirates) OR (emirate) AND ((‘2012’[Date – Publication]: ‘2022’[Date – Publication])) The search process complied with PRISMA guidelines. The study protocol was approved by PROSPERO (Protocol ID: CRD42022373179).

### Eligibility criteria

We applied predefined eligibility criteria to identify peer-reviewed studies examining PHI uptake and its association with healthcare utilization in GCC countries. The inclusion criteria and reasons for exclusion used during screening are summarized below.

### Inclusion criteria

- **Time frame**: 1 January 2012 to 31 October 2022.
- **Language**: English; full text available.
- **Participants**: Adults; studies assessing PHI status (enrolled vs. not enrolled) and/or comparing PHI with other coverage groups.
- **Place/Setting**: GCC countries (Saudi Arabia, UAE, Qatar, Oman, Kuwait, Bahrain).
- **Type of source**: Peer-reviewed original research articles.
- **Quality appraisal**: Included studies were required to meet a minimum methodological quality threshold based on CASP (≥50%).
- **Studies addressing**: (1) determinants of PHI uptake and/or (2) association between PHI and healthcare utilization.

### Reasons for exclusion criteria

- Outside time frame.
- Non-English and/or full text not accessible.
- Non-adult population and PHI not examined.
- Studies conducted outside the GCC; multi-country studies with no separate GCC results.
- Gray literature or non-original publications (e.g. conference abstracts/proceedings, theses/dissertations, books, and policy/NGO).
- Studies were excluded if they did not meet the minimum methodological quality threshold (CASP score <50%); however, no studies were excluded on this basis.
- Studies that do not report outcomes relevant to PHI uptake or healthcare utilization.

### Types of studies

All studies including qualitative, quantitative, or mixed-methods analyses were considered to be eligible for this review.

### Conditions or domains that have been studied

Private health insurance uptake and healthcare utilization.

### Selection of studies

The first reviewer, Khaled Althabaiti (KA), conducted the database search for relevant articles, using the pre-set search terms, which were refined based on training received in the university course ‘Systematic Reviews and Meta-analysis – SM00099’ at the University of Gothenburg. The second reviewer, Mohammad Badrul Bhuiyan (MBB), used the same search terms strategy. Both reviewers used Rayyan software for independent screening of the selected studies, following the inclusion and exclusion criteria for eligibility of the articles to be included in the review. The initial screening items included checking the titles and abstracts for relevance. The full texts of all included articles were then examined in parallel and separately by the two reviewers to determine whether they met all inclusion and exclusion criteria. Any disagreements between reviewers were discussed and resolved through dialogue. We had planned that if we faced any conflict, we would involve a third, independent reviewer to consult on the final decision. However, there was no conflict between the two primary reviewers. The full search strategy is provided in Supplementary Table S1, including the databases searched, the search terms used, the filters applied, the search period, and the initial number of studies identified. A total of 26 studies met the eligibility criteria and were included in the final review. A PRISMA flow diagram illustrating the study selection process is presented in Figure 3.

### Data extraction

Two reviewers (KA and MBB) independently extracted data into an Excel sheet. The following data were extracted for each study: title, year of publication, study aim, country studied, income category of that country, study design, data sources, and data collection methods, main findings of the study, any additional findings, conclusion, and study limitations. To ensure consistency, accuracy, and minimize bias in study selection, a third independent reviewer was involved in resolving any disagreements between the two primary reviewers regarding eligibility criteria. However, there was no conflict between the two primary reviewers. The details for each study are provided in Supplementary Table S2.

#### Quality assessment and Risk of bias assessment

We used the Critical Appraisal Skills Programme (CASP) checklists for both quantitative and qualitative studies, an effective tool for appraising the strengths and limitations of research designs [23]. The CASP checklist for quantitative studies provides a structured approach to assess the validity, outcomes, and relevance of quantitative studies. The checklist consists of 11 elements covering three main domains: (1) validity: This evaluates the study’s design and methods. Essential inquiries involve whether the research addressed a specific problem, whether the cohort was selected correctly to reduce bias, and whether the exposure and results were accurately measured. It is also essential to ascertain whether confounding factors were managed through approaches, such as stratification. (2) Outcomes: This segment evaluates the precision and dependability of results. Important factors include how the main findings are presented (e.g. risk ratios, odds ratios) and the accuracy of these findings as indicated by *p*-values, confidence intervals, and sample size. (3) Applicability: This section assesses the significance of outcomes for local situations. It evaluates whether the findings are trustworthy, relevant to local communities, and in agreement with other studies. The potential effects on clinical or public health practices should be evaluated to understand how the findings influence practical applications in the real world. Meanwhile, the checklist for the qualitative study included 10 elements that evaluated the design, research aims, methodology, recruitment, appropriateness of data collection, researcher–participant relationship, ethical considerations, data analysis, outcome, and value of the research.

**Figure 1.**
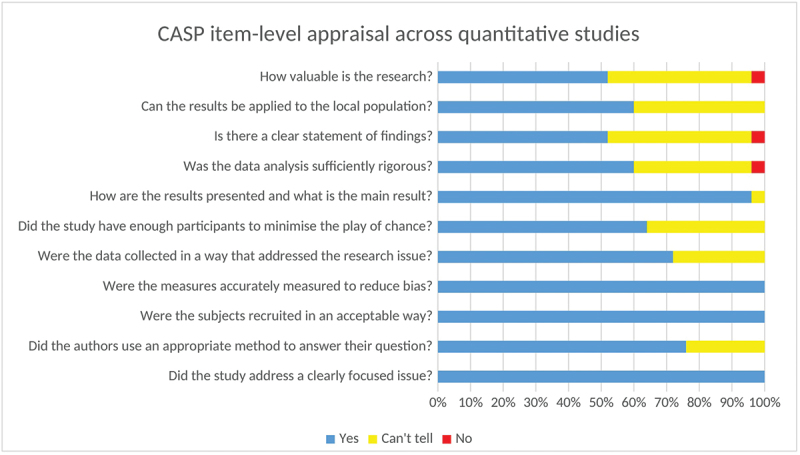
CASP item-level assessments across quantitative studies (*n* = 25).

**Figure 2.**
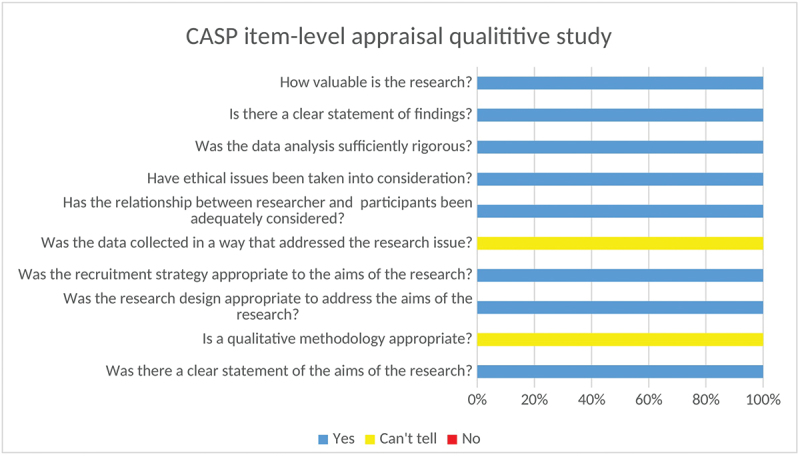
CASP item-level assessments for the included qualitative study (*n* = 1).

According to the assessment of each element, studies were rated as ‘Yes’ when the suggested requirement was fulfilled (score = 2), ‘Can’t tell’ when it was partially fulfilled (score = 1), or ‘No’ when it was absent or seriously deficient (score = 0). The CASP checklists were used to appraise and describe the methodological quality of the included studies. The CASP cross-sectional checklist was applied to 25 quantitative observational studies, while the CASP qualitative checklist was used for one qualitative study. Overall item-level CASP ratings across the included quantitative studies and the qualitative study are summarized in Figures 1 and 2. A pragmatic threshold of ≥50% (based on the total CASP score) was pre-specified by the review team to define eligibility; however, no studies were excluded because all included studies met this threshold. Detailed appraisal results for each study are presented in Supplementary Tables S3 (qualitative) and S4 (quantitative).

## Results

**Figure 3.**
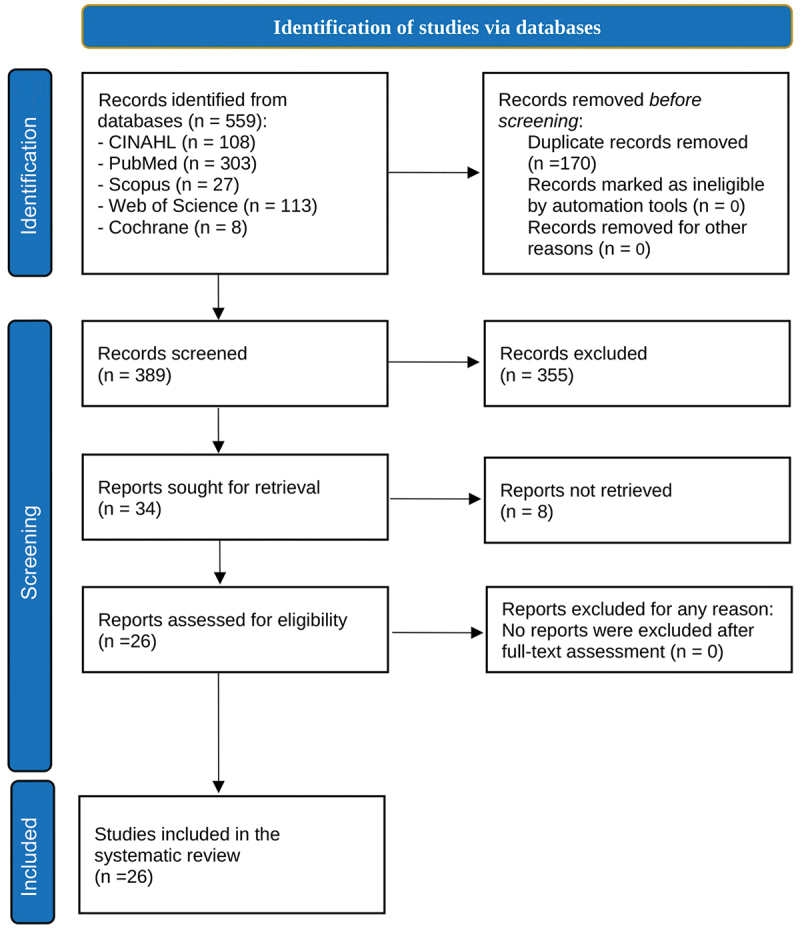
The PRISMA flow chart of the study selection process.

In total, 559 articles were identified from the CINAHL (*n* = 108), PubMed (*n* = 303), Scopus (*n* = 27), Web of Science (*n* = 113), and Cochrane Library (*n* = 8) databases (Figure 3). After removing duplicates (*n* = 170), the titles and abstracts of the remaining 389 articles were screened based on the inclusion and exclusion criteria. A total of 355 articles were excluded in the first stage, and 8 more articles were excluded due to a lack of full text. Overall, 26 articles were identified as eligible for this systematic review.

## General characteristics of the selected studies

Twenty-four of the 26 included articles used a cross-sectional design; one study was prospective, and one was retrospective. In total, 16 studies used primary data, and 10 used secondary data. Most studies were conducted in Saudi Arabia (*n* = 19), followed by the United Arab Emirates (*n* = 4) and Qatar (*n* = 2). One study examined GCC countries as a whole. Eleven articles investigated the determinants of PHI uptake, and 15 assessed the association between PHI and health service utilization (Table 1).

**Table 1.** Characteristics of the selected studies.

Characteristics	Frequency (%)
***Study design***	
Cross-sectional	24 (92.4)
Prospective study	1 (3.8)
Retrospective study	1 (3.8)
***Source of data***	
Primary	16 (61.5)
Secondary	10 (38.5)
***Impact outcome***	
Determinants of PHI uptake	11 (42.3)
Association between PHI and healthcare utilization	15 (57.7)
***Study setting country***	
All GCC countries	1 (3.8)
Saudi Arabia	19 (73.1)
United Arab Emirates	4 (15.4)
Qatar	2 (7.7)

Percentages are calculated based on the total number of included studies (*N* = 26).

### Determinants of private health insurance uptake in GCC countries

Willingness to pay was used as a proxy for demand for health insurance to explore potential determinants of interest in obtaining PHI in Saudi Arabia; it was measured as a binary (yes/no) response and reported as the proportion of participants willing to pay. Al-Hanawi et al. (2018) revealed that the proportion of participants reporting willingness to pay was influenced by household size, education, income, and satisfaction with public healthcare services [24]. Additionally, other studies showed that willingness to pay for PHI was higher among males, employed and married individuals, and those with chronic diseases [25], and older people [26]. Moreover, two of the included studies examined the quality of services; in Saudi Arabia, respondents were willing to pay for PHI to obtain higher quality services [25,27].

**Figure 4.**
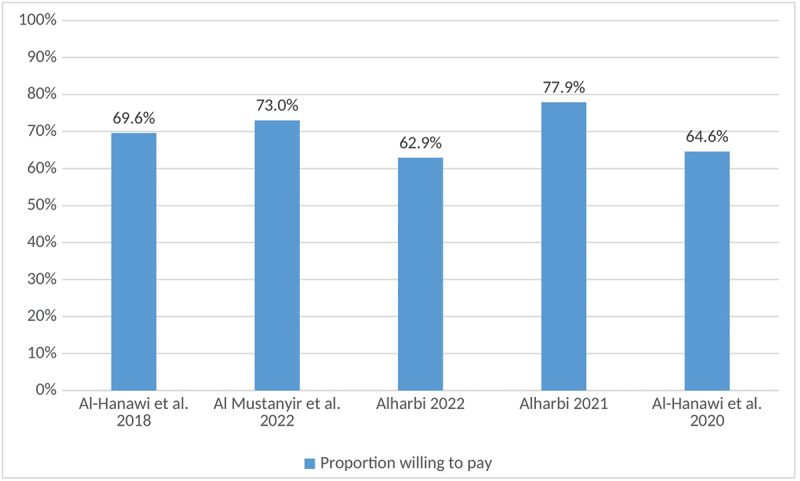
Proportion willing to pay for private health insurance: summary of included studies.

In Saudi Arabia, the proportion willing to pay for PHI among government school workers was found to be significantly higher among males, those with higher education levels and those diagnosed with chronic diseases [28]. In another study that explored the factors associated with PHI among expatriates, it was found that both personal and workplace characteristics are important factors that increase the likelihood of being an individual with PHI; these factors include being married and having a technical job that requires a high school level of education [29]. Studies in KSA showed that the proportion willing to pay for health insurance ranged from 62.9% to 77.9% [24–28], as summarized in Figure 4.

In KSA, the out-of-pocket (OOP) expenditure on chronic diseases was significantly higher among patients with PHI than among patients without PHI. This was partially explained by the notion that individuals often choose to purchase PHI based on their health-associated risks and their expectations of needing frequent health services [30]. In the same context, another study found that OOP expenditure was significantly higher among privately insured patients with an increased number of chronic diseases [31]. In another study in KSA, the decomposition of inequality in out-of-pocket health expenditures showed that the contributions of various socioeconomic factors to inequality were heterogeneously distributed between rich and poor individuals, such that OOP expenditures were generally higher among rich individuals than among poor individuals [15]. In another study covering all GCC countries, it was found that a considerable proportion of individuals without PHI had to borrow money to cover the costs of medical services [9]. In the United Arab Emirates, 79% of the randomly selected type 2 diabetic patients (T2DM) attending government hospitals had PHI, which indicated that having a chronic disease necessitating frequent visits to healthcare facilities encouraged patients to purchase PHI [32].

In summary, determinants of PHI uptake were mapped to Andersen’s Behavioral Model of Health Services Use (BMHSU) and categorized into three domains: (1) predisposing factors (e.g. sex, age, marital status, education), (2) enabling factors (e.g. employment/income and health system-related factors, such as access to services and perceived service quality), and (3) need factors (e.g. health status, including chronic conditions). These findings are summarized in Table 2, which presents the specific determinants identified across the included studies.

**Table 2.** Summary of studies on the determinants of private health insurance uptake in GCC countries.

Study	Country	Design	Sample size	Population	Determinants of uptake
Al-Hanawi et al. 2018	Saudi Arabia	Cross-sectional	1187	Saudi heads of household in Jeddah province	Smaller household size, higher education level, higher income, and satisfaction with healthcare services.
Al Mustanyir, Turner, and Mulcahy 2022	Saudi Arabia	Cross-sectional	600	General adult population in Riyadh	Gender, employment status, marital status, and having a chronic disease, and improvement of services
Alharbi 2021	Saudi Arabia	Cross-sectional	622	School workers	Higher education, chronic disease, and age
Al-Hanawi, Alsharqi, and Vaidya 2020	Saudi Arabia	Cross-sectional	1187	Heads of household in Jeddah Province	Conditional with improvement of services
(Alkhamis et al. 2017)	Saudi Arabia	Cross-sectional	4575	Male expatriate employees in the private sector (Riyadh)	Being married, occupying technical jobs, and having a higher level of education.
Almalki, Alahmari, Alqahtani, et al. 2022^a^	Saudi Arabia	Cross-sectional	1255	Households in Riyadh with members diagnosed with chronic NCDs	Chronic Non-Communicable Diseases
Almalki, Alahmari, Alqahtani, et al. 2022^b^	Saudi Arabia	Cross-sectional	1176	Households in Riyadh that used healthcare at least once in the past 3 months	Chronic Non-Communicable Diseases
Al-Hanawi, Mwale & Qattan, 2021	Saudi Arabia	Cross-sectional	8655	General population	Non-Saudi nationality, older age, male sex, married status, higher education, good self-rated health, and wealthier.
Alshamsan et al. 2017	GCC countries	Cross-sectional	3998	General population	Economic status
Radwan et al. 2020	United Arab Emirates	Cross-sectional	244	Diabetic patients	Chronic disease
Alharbi 2022	Saudi Arabia	Cross-sectional	475	General adult population in Saudi Arabia	Having a higher level of education and age

### Exploring the association between private health insurance and healthcare utilization in GCC countries

In KSA, nationally representative data were obtained from the Saudi Family Health Surveys (SFHS), and two studies were carried out. The first study revealed that PHI was positively associated with the likelihood of undergoing medical check-ups by 17%, compared with individuals without PHI. More specifically, there was a 13% increase in medical check-ups among citizens and a 20% increase among expatriates with PHI. These findings indicate that PHI encourages individuals to seek medical check-ups, even among citizens who have the privilege of using free services under the GHC [33]. The second study showed that individuals with PHI were more than twice as likely to seek periodic preventive health check-ups compared with those without PHI (OR = 2.29; 95% CI = 2.02–2.59; *p* ≤ 0.01) [17].

However, PHI did not necessarily increase the utilization of all healthcare facilities. Alsubaie et al. (2016), in a study conducted in KSA, concluded that the use of public primary healthcare at tertiary hospitals was much less common among individuals with PHI than among those without PHI. This was explained by the preference of individuals with PHI and with higher income to choose private health services over public facilities [34].

In the UAE, T2DM patients with PHI were two times more likely to use complementary and alternative medicine (CAM) than patients without PHI (OR: 2.08; CI: 1.11–3.86). Researchers argued that PHI by itself was not a direct predictor of CAM use, but this association could be explained by the fact that PHI holders, especially when employed, would have sufficient income to afford the OOP costs of CAM services [32].

Another study in the UAE highlighted that healthcare utilization under PHI varied by occupational class. The introduction of a single-payment system generated accurate data, which revealed discrepancies in the utilization of health services [35]. Malaviya et al. (2022) analyzed data from the Dubai Household Health Survey and found that PHI increased healthcare utilization among citizens and white-collar expatriates, but not among blue-collar laborers (e.g. construction, manual, and cleaning workers). This difference was attributed to less comprehensive PHI coverage benefits and additional barriers, such as limited awareness and demanding work conditions. Accordingly, having more comprehensive PHI coverage did not necessarily lead to higher utilization in this group, as utilization was also influenced by demographic factors, employment characteristics, and health awareness [36].

In KSA, researchers have found that a low understanding of the benefits of health insurance among expatriates who had PHI was associated with less access to healthcare services (OR = 0.393, CI = 0.335–0.461), which highlights the importance of expatriates’ knowledge about the benefits of PHI rather than the details of numerical copays [37]. In contrast, another study in Saudi Arabia found that adult patients with chronic diseases who had PHI were more likely to revisit emergency departments (ED) compared with those without PHI (aRR = 4.23, 95% CI: 2.60–6.90; *p* = 0.001) [38].

Additionally, studies in both KSA and the UAE reported that PHI was associated with improved adherence to long-term treatment. In a university hospital in Saudi Arabia, patients with PHI had significantly higher adherence scores to antiplatelet therapy (33 ± 4.5) than those with GHC (30.6 ± 3.4) [39]. Similarly, in the United Arab Emirates, it was shown that adherence to treatment for multimorbidity was significantly higher among patients with PHI (77.5%) [40].

A study in KSA found that the number of prescribed medicines (mean = 3.37 ± SD = 2.10) and their prices (mean = 460 SR ± SD = 387.3) were significantly higher among individuals with PHI compared with those without [41]. However, a survey of 16 insurance companies to investigate their policies regarding pharmaceutical coverage revealed that there was marked inconsistency in the regulations regarding the maximum time of treatment and allowed refill of treatment for patients with PHI [42]. From another perspective, a study in Saudi Arabia found that PHI was associated with a paradoxical effect on out-of-pocket expenditures among individuals with PHI across socioeconomic statuses. While there was a reduction in health and medicine expenditures among low-income patients, the opposite occurred as income increased. Researchers have claimed that healthcare providers benefit from high-income patients by prescribing medicine that is not covered by PHI [43].

In Qatar, all types of PHI were associated with higher inpatient and outpatient service utilization for both citizens and expatriates compared with those without any form of PHI, even after controlling for other factors, such as age, gender, and marital status, which indicated that improving PHI could promote greater utilization of care [44].

In KSA, one study compared individuals with PHI and those without PHI in terms of the quality and fairness of health services in a tertiary hospital; the results revealed that the patients with PHI rated their services as high quality and fair, while the patients without PHI had been subjected to prolonged waiting periods, payment procedures, and differences in treatment [45]. On the other hand, a study conducted in Qatar found no statistically significant difference in overall satisfaction between individuals with PHI and those without. While a study from KSA indicated higher satisfaction among PHI beneficiaries, the findings from Qatar suggest no significant difference. This inconsistency may be due to variations in the benefit package or quality of services provided by PHI, which can differ among countries [46].

PHI was positively associated with health service utilization, particularly check-ups, periodic preventive services, inpatient and outpatient services, ED visits, and medication dispensing. Moreover, PHI was positively associated with the behavior and attitudes of beneficiaries, as indicated by the increased adherence to treatment and positive perceptions about the quality of received services. In addition, PHI was positively associated with higher utilization of complementary and alternative medicines and increased out-of-pocket expenditures on medicines, especially among highly wealthy individuals. However, higher utilization among PHI holders does not necessarily indicate improved equity. Several included studies suggest that the utilization gains under PHI may be concentrated among higher-income or better-covered groups, whereas low-income or low-literacy workers may face benefit limitations and informational barriers, which can widen socioeconomic differences in access and use [34,36,37]. Table 3 summarizes the evidence from included studies showing that PHI is positively associated with increased healthcare utilization.

**Table 3.** Summary of studies on the association between private health insurance and healthcare service utilization.

Study	Country	Design	Population (*n*)	Type of service	Association with PHI*	Effect size	Effect size metric**
Al-Hanawi et al. 2020	Saudi Arabia	Cross-sectional	National adult sample from SFHS 2018 (*n* = 8845)	Medical checkup	Positive	↑ 17%	Percentage
Al-Hanawi and Chirwa 2021	Saudi Arabia	Cross-sectional	National adult sample from SFHS 2018 (*n* = 11,528)	Periodic preventive services	Positive	OR = 2.29; 95% CI = 2.02–2.59	Odds ratio
Alsubaie et al. 2016	Saudi Arabia	Cross-sectional	Tertiary hospital PHC attendees, Riyadh (*n* = 358)	Visits to primary healthcare	Positive	(OR = 8.333; 95% CI = 1.037–66.667, *p* = 0.046)	Odds ratio
Radwan et al. 2020	United Arab Emirates	Cross-sectional	T2DM outpatients in two governmental hospitals (Dubai and Sharjah), *n* = 244	Use complementary and alternative medicine in T2DM	Positive	OR: 2.08; CI: 1.11–3.86	Odds ratio
Malaviya et al. 2022	United Arab Emirates	Cross-sectional	Dubai Household Health Survey in 2014 and 2018 (households: 3271 in 2014; 2200 in 2018)	Inpatients and outpatient services	Positive	↑ 80% from 2014 to 2018	Percentage
Alkhamis 2018	Saudi Arabia	Cross-sectional	Male expatriates with PHI in Riyadh (*n* = 3398)	Lack of access to healthcare (past 1 year)	Negative association with knowledge level	OR = 0.393, CI = 0.335–0.461	Odds ratio
Anwar E. Ahmed et al. 2018	Saudi Arabia	Retrospective study	Adults with chronic diseases at KAMC adult ED, Riyadh (*n* = 24,206)	Revisits of chronic diseases’ adult patients to emergency department	Positive	aRR = 4.23, CI: 2.60–6.90	Adjusted relative rate
Liu et al. 2020	Qatar	Cross-sectional	Adults reporting illness in the Household Utilization and Expenditure Survey 2014 (*n* = 3016)	Inpatients and outpatient services	Positive	↑ 11% for citizens and 22% for expatriates	Percentage
Alrabiah et al. 2020	Saudi Arabia	Cross-sectional	Adults with acute coronary syndrome receiving dual antiplatelet therapy in a cardiac outpatient clinic, Riyadh (*n* = 192)	Adherence to prophylactic treatment	Positive	PHI SEAMS = (33 ± 4.5)	Mean ± SD
Allaham et al. 2022	United Arab Emirates	Cross-sectional	Adults with ≥2 chronic diseases attending outpatient clinics in two tertiary hospitals in Dubai (*n* = 630)	Adherence to multimorbidity treatment	Positive	77.5% in privately insured	Percentage
Al-Mohamadi et al. 2014	Saudi Arabia	Prospective study	Patients with chronic diseases in three private hospitals, Jeddah (*n* = 363)	Number of prescribed medications and total prescription cost	Positive (patients with PHI received more and higher cost medications)	Number of prescribed items was higher among patients with PHI than without PHI patients (6.82 ± 3.23 vs. 4.84 ± 2.70), and the total prescription cost was also higher for those with PHI (638 ± 386.6 vs. 460 ± 387.3 SAR)	Mean ± SD
Bawazir et al. 2013	Saudi Arabia	Cross-sectional	PHI companies (16 companies)	Pharmaceutical coverage policies (maximum treatment duration and refill limits)	Not applicable (policy-level variability in PHI benefits)	Differences in treatment duration and refill rules between PHI companies	Not applicable
(Al-Hanawi, Mwale, and Qattan 2021	Saudi Arabia	Cross-sectional	Nationally representative sample from the 2018 SFHS (*n* = 8,655)	Out-of-pocket expenditure on health and medicines	Mixed pattern	PHI reduces OOP spending on health by 3.6% and on medicines by 5.2%, but OOP slightly increases with higher income among PHI holders (+0.4% for health and +0.5% for medicines).	Percentage
Binsaeed et al. 2022	Saudi Arabia	Cross-sectional	Adults attending a public tertiary hospital in Riyadh (*n* = 350)	Quality of services	Positive	(Mean perceived quality = 3.37, SD = 0.525) in privately insured, and (3.06, SD = 0.452, *p* 0.001) in uninsured	Mean ± SD
Khaled, Shockley, and Abdul Rahim 2017	Qatar	Cross-sectional	Nationally representative adults ≥18 years with satisfaction data (*n* = 2751)	Satisfaction about health services	No significant association	OR = 1.65, CI = 0.97–2.82, *p* = 0.20	Odds ratio

*‘Positive’ indicates higher utilization among individuals with PHI compared with those without PHI; ‘negative’ indicates lower utilization.

Table 4 summarizes the categorization of the determinants of PHI uptake and healthcare utilization patterns based on the Andersen’s BMHSU, providing a structured framework to interpret how predisposing, enabling, and need factors interact to shape patterns of access and service use across the included studies

**Table 4.** Mapping of identified determinants and healthcare utilization patterns to Andersen BMHSU categories.

Study	Focus Area (Uptake/Utilization)	Finding	Andersen Model Category	Classification Rationale
Al-Hanawi et al. 2018	Uptake	Willingness to pay for obtaining PHI is influenced by higher education level, higher income, household size, and type of healthcare financing	Predisposing + enabling	Sociodemographic characteristic (predisposing); healthcare financing and satisfaction (enabling)
Al Mustanyir, Turner, and Mulcahy 2022	Uptake	Willingness to pay for obtaining PHI associated with higher among males, employed, married, older adults, and those with chronic conditions	Predisposing + enabling + need	Demographics (predisposing); perceived need for higher quality healthcare services (enabling); chronic disease (need)
Alharbi 2022	Uptake	Willingness to pay for obtaining National Health Insurance was positively associated with higher education, male gender, older age, higher income, and previous insurance experience	Predisposing	Although focused on NHI, which was not started in Saudi Arabia, the study measured Willingness to pay as a proxy for potential interest in PHI. The identified determinants reflect socioeconomic and demographic factors relevant to PHI uptake decisions
Alharbi 2021	Uptake	Government school workers’ Willingness to pay was higher among males, the educated, and the chronically ill	Predisposing + Need	Gender and education (predisposing); health condition (need)
Al-Hanawi, Alsharqi, and Vaidya 2020	Uptake	Willingness to pay for obtaining PHI, driven by a perceived need for higher-quality healthcare services and improved public healthcare, was associated with male gender, higher income, higher education, and older age	Predisposing + enabling	Gender, age, education (predisposing); income, dissatisfaction, and PHI (enabling)
Alkhamis et al. 2017	Uptake	PHI is more likely among married expatriates with technical jobs and higher education	Predisposing	Marital and occupational status reflect demographic predisposition
Almalki, Alahmari, Alqahtani et al. 2022	Uptake	Out-of-pocket (OOP) spending was significantly higher among individuals with PHI who had multiple chronic conditions, likely due to increased healthcare needs and service utilization	Need + enabling	Chronic illness reflects objective need; the PHI holder indicates enabling access and financial capacity
Almalki, Alahmari, Alqahtani et al. 2022	Uptake	OOP spending was significantly higher among PHI holders with multiple chronic conditions, likely due to the anticipated need for frequent healthcare	Need + enabling	Chronic illness reflects need. Holding PHI indicates an enabling factor that facilitates access to healthcare
Al-Hanawi et al. 2021	Uptake	Inequality in OOP expenditure related to income differences among PHI holders	Enabling	Income level affects both access and may influence individuals’ perceptions of the value and adequacy of PHI and affect future enrollment decisions
Alshamsan et al. 2017	Uptake	Individuals without PHI had to borrow for healthcare	Enabling	Lack of financial resources influences the inability to enroll in PHI
Radwan et al. 2020	Uptake	79% of T2DM patients had PHI	Need	Chronic disease status motivates insurance uptake
Al-Hanawi et al. 2020	Utilization	PHI was positively associated with the likelihood of seeking medical check-ups by 17%	Enabling	PHI coverage facilitates access to preventive care
Al-Hanawi and Chirwa 2021	Utilization	PHI associated with higher periodic preventive check-ups (OR = 2.29)	Enabling	PHI facilitates access to routine care, increasing utilization
Alsubaie et al. 2016	Utilization	PHI holders are less likely to use public PHC; prefer private healthcare	Enabling	PHI reduces cost burden, encouraging service use
Radwan et al. 2020	Utilization	T2DM patients with PHI more likely to use CAM (OR: 2.08)	Enabling + predisposing	PHI holders associated with employed individuals can afford CAM based on income and job class
Malaviya et al. 2022	Utilization	PHI increased utilization among citizens and white-collar expatriates but not among blue-collar laborers; differences attributed to demographic and occupational class	Enabling + predisposing	Occupational class and demographic profile (e.g. younger, healthier blue-collar workers) reflect predisposing factors; PHI coverage reflects enabling factor, though effect varies by socioeconomic subgroup
Alkhamis 2018	Utilization	Poor knowledge of PHI benefits linked to lower access (OR = 0.393)	Enabling	Knowledge about PHI serves as an enabling factor that facilitates informed access and effective use of healthcare services
Anwar E. Ahmed et al. 2018	Utilization	PHI holders with chronic diseases were significantly more likely to revisit emergency departments compared with non-PHI holders (RR = 4.23)	Need + enabling	Chronic disease represents a need factor; PHI coverage serves as an enabling factor that facilitates access to emergency care
Anwar E. Ahmed et al. 2018	Utilization	KSA and UAE: PHI associated with better adherence to long-term treatment	Need + enabling	Chronic disease represents a need factor; PHI coverage serves as an enabling factor that facilitates access to emergency care
Alrabiah et al. 2020	Utilization	Patients with PHI had significantly higher adherence to antiplatelet therapy compared to those with GHC	Enabling	PHI coverage facilitates consistent access to medications and follow-up care, supporting long-term treatment adherence
Allaham et al. 2022	Utilization	Adherence to treatment for multimorbidity was significantly higher among patients with PHI (77.6%)	Enabling	PHI improves access to needed medications and continuity of care
Al-Mohamadi et al. 2014	Utilization	The number of prescribed medicines (mean difference = 3.37 ± SD = 2.10) and their prices (mean difference = 460 SR ± SD = 387.3) were significantly higher among individuals with PHI compared to those without	Enabling	PHI facilitates greater access to medications, potentially leading to more prescriptions and higher pharmaceutical expenditures
Bawazir et al. 2013	Utilization	Differences in PHI plans’ treatment duration and refill rules may limit access to needed medications	Enabling	PHI policy terms affect access to continuous medication and treatment adherence
Al-Hanawi MK 2021	Utilization	PHI had a paradoxical effect on OOP spending: reduced among low-income individuals, but increased among high-income PHI holders, possibly due to uncovered prescriptions	Enabling	Socioeconomic status and PHI coverage influence financial access and provider behavior, affecting actual healthcare costs and service utilization
Liu et al. 2020	Utilization	PHI was associated with higher utilization of inpatient and outpatient services among both citizens and expatriates	Enabling	PHI facilitates access to healthcare services and removes financial and administrative barriers to utilization
Binsaeed et al. 2022	Utilization	PHI holders perceived higher quality and fairness in services, while non-PHI patients reported longer wait times, payment burdens, and treatment disparities	Enabling	Having PHI facilitates access to more efficient and equitable care, influencing patients’ experiences and satisfaction with service quality

## Discussion

In this systematic review, we found that sociodemographic and socioeconomic characteristics, such as gender, age, marital status, education level, employment status, and monthly income, were consistently associated with PHI uptake. Regarding healthcare utilization, most included studies reported that having PHI was associated with higher healthcare utilization compared to those without PHI, although one study found a negative association.

Variability in the reported determinants across studies may partly reflect evolving health policies and eligibility criteria over the past decade. In particular, the regional transition from universal public health coverage for all residents to a model that provides free care primarily to citizens and selected expatriate groups, alongside the introduction of mandatory PHI for private-sector expatriates, has fundamentally reshaped the determinants of insurance enrollment and service use. This policy shift has created a clearer and more stratified context for examining how individuals engage with healthcare services under differing insurance regimes [2,9,10].

According to Andersen’s BMHSU, healthcare utilization is determined by three interrelated categories of factors that collectively shape access and use of care. Predisposing factors refer to the demographic and attitudinal characteristics of individuals; enabling factors capture the financial and structural resources, such as health insurance status and service availability that facilitate access to care; and need factors represent both the perceived and objectively assessed health requirements that drive healthcare utilization [47]. In line with the BMHSU (1973), the current review demonstrated that sociodemographic characteristics represented the predisposing factor, while health status (for instance, chronic disease) represented the ‘need,’ and quality of healthcare services represented the ‘enabling’ factor. Similar findings were recognized in another systematic review carried out in Iran, which revealed that healthcare utilization was influenced by the sociodemographic variables of the household, health status, and type of health services [48].

Several included studies revealed that PHI had a positive association with the utilization of health services, especially check-up services, periodic preventive services, in-patient and outpatient services, visits to the ED, and dispensing medicines. Moreover, these findings highlight the need to explore possible mechanisms through which PHI encourages utilization (e.g. reduced financial barriers and high-quality services). However, the generalizability of these findings is limited by variations in study populations and the types of health services assessed across the included studies [17,49–52]. This pattern aligns with Andersen’s BMHSU, suggesting that PHI can be understood as an enabling factor that facilitates access to healthcare by reducing financial barriers and enhancing service availability. Nevertheless, differences in benefit coverage, employment type, and income levels across GCC countries may shape the extent to which PHI improves healthcare utilization, raising important considerations for equity within the region’s ongoing health system reforms.

Chronic noncommunicable diseases are a leading factor in the utilization of healthcare services. Chronic diseases require a long period of treatment with frequent visits to healthcare facilities, resulting in an extra burden on the health system as well as the patients [53]. In the United States, the direct cost of treating chronic disease patients totaled 1.1 trillion dollars in 2003, and the indirect cost of economic productivity is projected to reach $3.7 trillion in 2023 [54]. Patients who are the customers of healthcare services have expectations for the quality of service that warrant satisfaction [55]; therefore, as anticipated, two of the reviewed studies reported that the willingness to pay for PHI was conditional on the improved quality of health services [25,27]. Within Andersen’s BMHSU, chronic illness represents a ‘need’ factor that strongly drives healthcare utilization, while PHI can function as an enabling factor that mitigates the financial burden of long-term treatment. In the GCC context, where the prevalence of chronic diseases is rapidly increasing, ensuring financial protection through comprehensive PHI coverage aligns with ongoing health system reforms aimed at achieving UHC [56].

One notable issue identified in this review is the disparities in healthcare usage between expatriates and citizens, specifically among low-income migrant workers, which reflects unequal access to and use of health services. PHI is a requirement for expatriates throughout the GCC, but various studies show that low-income or less educated expatriates tend to receive limited benefit coverage, with restricted provider networks, and at the same time have insufficient knowledge of their rights [3,36]. Therefore, this group tends to underutilize preventive and chronic care services, which may contribute to disparities in healthcare utilization. From Andersen’s perspective, these disparities reflect inequalities in enabling factors, such as financial resources and health literacy. Such structural gaps may hinder progress toward equity within ongoing GCC health reforms, underscoring the need for more inclusive and equitable PHI policies.

While the current study revealed that knowledge about the benefits of PHI and perceptions of high-quality service are associated with a greater likelihood of enrollment in PHI, Dror et al. (2016) described these factors as enablers in addition to trust in scheme management [57]. Knowledge about PHI could be viewed as a crucial factor in determining enrollment in PHI in Gulf States, due to the relatively high percentage of expatriates who mainly originate from different countries worldwide, particularly from low-income countries with diverse cultures and health systems. This knowledge may include understanding what healthcare services are covered, how to access care, and how to file a claim [57]. Since PHI enrollment is mandatory for expatriates in the GCC countries, the primary concern shifts from initial uptake to ensuring adequate awareness and understanding of the utilization of PHI benefits. In addition, Dror et al. (2016) identified the factors that encourage the renewal of PHI, including the receipt of an insurance payout the previous year, and the barriers to renewal, which included (a) stringent rules of some Community-Based Health Insurance (CBHI) schemes, (b) inadequate legal and policy frameworks to support CBHI and (c) limited or inappropriate benefits packages [57].

This systematic review provided policymakers and healthcare providers in GCC countries with actionable insights on how PHI can complement national health financing schemes within UHC initiatives. These insights include strengthening regulation of employer-based coverage, defining minimum benefit standards that include primary and preventive care, and improving insurance literacy to support appropriate and equitable healthcare utilization, particularly among low-income expatriates.

## Limitations of the study

This study has several limitations. First, the availability of published research was uneven across GCC countries, with some countries having more studies than others. In particular, most of the included studies were conducted in KSA (19 out of 26). This uneven distribution limited our ability to conduct balanced comparisons between these countries. In addition, smaller GCC countries may differ from KSA in labor markets and PHI regulation, which may bias the findings and limit their generalizability across the GCC region. Second, the study populations across the reviewed articles were different, thereby potentially impacting the generalizability of the findings. Third, the types of investigated health services were inconsistent, thus making direct comparisons challenging. Fourth, because ‘health insurance’ is a broad term, some misclassification is possible; however, we screened studies to ensure that coverage corresponded to private health insurance. Lastly, due to the limited number of studies and heterogeneity in the types of health services examined, we were unable to perform a meta-analysis. Additionally, the exclusion of gray literature may have introduced publication bias.

## Conclusions and recommendations

The growing challenges facing healthcare systems worldwide have led the GCC countries to adopt reforms aimed at ensuring the sustainability of UHC. Although GHC has long provided broad access to care, the increasing population diversity and rising healthcare demand have prompted the integration of PHI as a complementary mechanism. The current study highlights the role of PHI in increasing health service utilization, which will help improve access and system efficiency during the current period of transition toward UHC.

The factors associated with PHI uptake included male sex, older age, being married, higher educational level, a high monthly income, and chronic disease. Eventually, PHI was positively associated with increased utilization of healthcare services, particularly check-up services, periodic preventive services, inpatient and outpatient services, and ED visits, as well as increased dispensing of medications. The willingness of people in GCC countries to pay for PHI was conditional on improved quality, which will influence the efficiency of health service utilization. Although PHI is mandatory for expatriate workers in GCC countries, those with low-income and limited education often receive minimal coverage and may lack awareness of how to fully utilize their insurance benefits.

We recommend that governments and regulators, who hold the primary responsibility for addressing health inequalities and unmet needs, require PHI companies, in collaboration with employers, to develop tailored insurance policies for expatriate workers with low-income and limited health awareness, as these groups are less likely to engage in preventive healthcare. Such collaboration could include orientation workshops and the distribution of simplified multilingual booklets to enhance awareness of entitlements, claims procedures, and available benefits. Moreover, policymakers should consider incentives or subsidies for employers who provide high-quality PHI to their employees. PHI policies should also establish clear and transparent claims mechanisms and grievance redressal systems that are accessible to individuals with limited administrative literacy.

## Supplementary Material

PRISMA_2020_checklist.docx

Supplementary_Table_S2_Data_extraction.xlsx

Supplementary_Table_S3_Quality assessment_qualitative_studies.xlsx

Supplementary_Table_S4_Quality assessment_quantitative studies.xlsx

Supplementary_Table_S1_Search_Strategy.docx

## Data Availability

The datasets supporting the conclusions and used for the review article are included within the article and its additional files.
