# Supplementary material for: Determinants of private health insurance uptake and its association with healthcare utilization in Gulf Cooperation Council countries: a systematic review
Source: Glob Health Action. 2026 Mar 25;19(1):2647528. doi: 10.1080/16549716.2026.2647528 (PMC13021022; doi:10.1080/16549716.2026.2647528)
Supplement: PRISMA_2020_checklist.docx [file ZGHA_A_2647528_SM3521.docx]

| **Section and Topic** | **Item #** | **Checklist item** | **Location where item is reported** |
| --- | --- | --- | --- |
| **TITLE** | | |  |
| Title | 1 | Identify the report as a systematic review. | \| p.1 (Title page) \| \| --- \|  \|  \| \| --- \| |
| **ABSTRACT** | | |  |
| Abstract | 2 | See the PRISMA 2020 for Abstracts checklist. | p.2 (Abstract) |
| **INTRODUCTION** | | |  |
| Rationale | 3 | Describe the rationale for the review in the context of existing knowledge. | p.3–4 (Introduction) |
| Objectives | 4 | Provide an explicit statement of the objective(s) or question(s) the review addresses. | p.5 (Introduction – last paragraph) |
| **METHODS** | | |  |
| Eligibility criteria | 5 | Specify the inclusion and exclusion criteria for the review and how studies were grouped for the syntheses. | p.5-6 (Methods – Eligibility criteria) |
| Information sources | 6 | Specify all databases, registers, websites, organisations, reference lists and other sources searched or consulted to identify studies. Specify the date when each source was last searched or consulted. | p.5–6 (Methods – Information sources) |
| Search strategy | 7 | Present the full search strategies for all databases, registers and websites, including any filters and limits used. | p.6–8 (Methods – Search strategy and Table 2) |
| Selection process | 8 | Specify the methods used to decide whether a study met the inclusion criteria of the review, including how many reviewers screened each record and each report retrieved, whether they worked independently, and if applicable, details of automation tools used in the process. | p.7 (Methods – Selection of Studies) |
| Data collection process | 9 | Specify the methods used to collect data from reports, including how many reviewers collected data from each report, whether they worked independently, any processes for obtaining or confirming data from study investigators, and if applicable, details of automation tools used in the process. | p.9 (Methods – Data extraction) |
| Data items | 10a | List and define all outcomes for which data were sought. Specify whether all results that were compatible with each outcome domain in each study were sought (e.g. for all measures, time points, analyses), and if not, the methods used to decide which results to collect. | p.7(domains studied) |
|  | 10b | List and define all other variables for which data were sought (e.g. participant and intervention characteristics, funding sources). Describe any assumptions made about any missing or unclear information. | p.9 (Methods – Data extraction variables; No assumptions were made regarding missing data) |
| Study risk of bias assessment | 11 | Specify the methods used to assess risk of bias in the included studies, including details of the tool(s) used, how many reviewers assessed each study and whether they worked independently, and if applicable, details of automation tools used in the process. | p.9-10 (Methods – Quality Assessment & Risk of Bias; CASP checklist used; two independent reviewers; no automation tools) |
| Effect measures | 12 | Specify for each outcome the effect measure(s) (e.g. risk ratio, mean difference) used in the synthesis or presentation of results. | Not applicable |
| Synthesis methods | 13a | Describe the processes used to decide which studies were eligible for each synthesis (e.g. tabulating the study intervention characteristics and comparing against the planned groups for each synthesis (item #5)). | p.7 (Methods – Selection of Studies and Eligibility criteria) |
|  | 13b | Describe any methods required to prepare the data for presentation or synthesis, such as handling of missing summary statistics, or data conversions. | Not applicable |
|  | 13c | Describe any methods used to tabulate or visually display results of individual studies and syntheses. | p.11–21 (Tables 3–6; Results section) |
|  | 13d | Describe any methods used to synthesize results and provide a rationale for the choice(s). If meta-analysis was performed, describe the model(s), method(s) to identify the presence and extent of statistical heterogeneity, and software package(s) used. | p.14–21 (Results and Discussion – narrative synthesis based on Andersen’s Behavioral Model) |
|  | 13e | Describe any methods used to explore possible causes of heterogeneity among study results (e.g. subgroup analysis, meta-regression). | p. 14 – 21 (Results and Discussion sections) |
|  | 13f | Describe any sensitivity analyses conducted to assess robustness of the synthesized results. | p. 9 and 14–21 (Quality Assessment and Discussion sections) |
| Reporting bias assessment | 14 | Describe any methods used to assess risk of bias due to missing results in a synthesis (arising from reporting biases). | No formal assessment of reporting bias was conducted due to the absence of meta-analysis. Potential bias was minimized through a comprehensive multi-database search and clear inclusion criteria. |
| Certainty assessment | 15 | Describe any methods used to assess certainty (or confidence) in the body of evidence for an outcome. | Not formally assessed; study quality evaluated using CASP. |
| **RESULTS** | | |  |
| Study selection | 16a | Describe the results of the search and selection process, from the number of records identified in the search to the number of studies included in the review, ideally using a flow diagram. | Reported in Results section (p. 11) and Figure 1 (PRISMA flow diagram). |
|  | 16b | Cite studies that might appear to meet the inclusion criteria, but which were excluded, and explain why they were excluded. | No specific excluded studies were cited; all exclusions summarized in Figure 1 (PRISMA flow diagram). |
| Study characteristics | 17 | Cite each included study and present its characteristics. | Described in Data Extraction (p. 9) and summarized in Table 3 (p. 12). |
| Risk of bias in studies | 18 | Present assessments of risk of bias for each included study. | Risk of bias assessed using CASP checklists (p. 9). |
| Results of individual studies | 19 | For all outcomes, present, for each study: (a) summary statistics for each group (where appropriate) and (b) an effect estimate and its precision (e.g. confidence/credible interval), ideally using structured tables or plots. | Results of individual studies are summarized in Tables 4 and 5 (p. 13–15). |
| Results of syntheses | 20a | For each synthesis, briefly summarise the characteristics and risk of bias among contributing studies. | Characteristics and quality of included studies summarized in Table 3 (p. 12) and assessed using CASP (p. 9). |
|  | 20b | Present results of all statistical syntheses conducted. If meta-analysis was done, present for each the summary estimate and its precision (e.g. confidence/credible interval) and measures of statistical heterogeneity. If comparing groups, describe the direction of the effect. | Not applicable |
|  | 20c | Present results of all investigations of possible causes of heterogeneity among study results. | Variations across studies discussed in the Discussion section (p. 16–17). |
|  | 20d | Present results of all sensitivity analyses conducted to assess the robustness of the synthesized results. | Not applicable |
| Reporting biases | 21 | Present assessments of risk of bias due to missing results (arising from reporting biases) for each synthesis assessed. | Not applicable |
| Certainty of evidence | 22 | Present assessments of certainty (or confidence) in the body of evidence for each outcome assessed. | Not applicable |
| **DISCUSSION** | | |  |
| Discussion | 23a | Provide a general interpretation of the results in the context of other evidence. | General interpretation of findings provided in the Discussion section (p. 16–18), comparing with previous evidence. |
|  | 23b | Discuss any limitations of the evidence included in the review. | Limitations of included evidence (e.g., varied study designs, populations, and self-reported data) discussed in the Limitations section. |
|  | 23c | Discuss any limitations of the review processes used. | Limitations of the review process (e.g., exclusion of grey literature and no meta-analysis due to heterogeneity) discussed in the Limitations section. |
|  | 23d | Discuss implications of the results for practice, policy, and future research. | Implications for practice, policy, and research were discussed, emphasizing equitable PHI reform and the need for future studies assessing post-reform effects. |
| **OTHER INFORMATION** | | |  |
| Registration and protocol | 24a | Provide registration information for the review, including register name and registration number, or state that the review was not registered. | The review was registered in PROSPERO (ID: CRD42022371719). |
|  | 24b | Indicate where the review protocol can be accessed, or state that a protocol was not prepared. | The review protocol can be accessed on the PROSPERO website (registration ID: CRD42022371719). |
|  | 24c | Describe and explain any amendments to information provided at registration or in the protocol. | No amendments were made to the registered protocol. |
| Support | 25 | Describe sources of financial or non-financial support for the review, and the role of the funders or sponsors in the review. | No specific funding; conducted as part of a PhD. |
| Competing interests | 26 | Declare any competing interests of review authors. | The authors declare no competing interests. |
| Availability of data, code and other materials | 27 | Report which of the following are publicly available and where they can be found: template data collection forms; data extracted from included studies; data used for all analyses; analytic code; any other materials used in the review. | Data available upon reasonable request. |

*From:*  Page MJ, McKenzie JE, Bossuyt PM, Boutron I, Hoffmann TC, Mulrow CD, et al. The PRISMA 2020 statement: an updated guideline for reporting systematic reviews. BMJ 2021;372:n71. doi: 10.1136/bmj.n71. This work is licensed under CC BY 4.0. To view a copy of this license, visit <https://creativecommons.org/licenses/by/4.0/>
