# Supplementary material for: Determinants of private health insurance uptake and its association with healthcare utilization in Gulf Cooperation Council countries: a systematic review
Source: Glob Health Action. 2026 Mar 25;19(1):2647528. doi: 10.1080/16549716.2026.2647528 (PMC13021022; doi:10.1080/16549716.2026.2647528)
Supplement: Supplementary_Table_S1_Search_Strategy.docx [file ZGHA_A_2647528_SM3517.docx]

**Supplementary Table S1: Description of the search strategy employed in the searched databases.**

| **Database** | **Search String** | **Limits** | **Date** | **Number of Retrieved Studies** |
| --- | --- | --- | --- | --- |
| PubMed | (((((((("insurance, health"[MeSH Terms]) OR (health insurance*[tiab])) OR ("insured"[Title/Abstract])) OR ("uninsured"[Title/Abstract])) OR ("community insurance"[Title/Abstract])) OR ("medical insurance [Title/Abstract])) OR ("universal healthcare"[Title/Abstract])) AND (((((((((((GCC) OR (Gulf council cooperation)) OR (Bahrain*)) OR (Saudi)) OR (KSA)) OR (Kuwait*)) OR (Oman*)) OR (Qatar*)) OR (UAE)) OR (United Arab Emirates)) OR (emirate)))) AND (("2012 [Date - Publication] : "2022"[Date - Publication])) | English,10 years | 01/01/2012 to 31/10/2022 | 303 |
| Cochrane Library | #1 insurance, health :MeSH #2 ("insured" OR "uninsured" OR "community insurance" OR "medical insurance" OR "universal healthcare" OR "Health insurance"):ti,ab,kw  #3 #1 OR #2 #4 (GCC* OR Bahrain* OR Saudi* OR KSA* OR Kuwait* OR Oman* OR Qatar* OR UAE* OR United Arab Emirates* OR emirate*):ti,ab,kw  #5 #3 AND #4 with Cochrane Library publication date Between Jan 2012 and Oct 2022 | English,10 years | 01/01/2012 to 31/10/2022 | 8 |
| Scopus | ((health AND insurance)) OR ((insured)) OR ((uninsured)) OR ((community AND insurance)) OR ((medical AND insurance)) OR ((health AND coverage)) AND ((saudi AND arabia OR kingdom AND of AND saudi AND arabia OR ksa OR uae OR gcc OR gulf OR united AND arab AND emirates OR kuwait OR oman OR qatar)) | English,10 years | 01/01/2012 to 31/10/2022 | 27 |
| Web of Science | (("Health Insurance") or ("insured") Or ("uninsured") Or ("community insurance") Or ("medical insurance") Or ("health coverage")) And (Saudi Arabia or Bahrain or Kuwait or GCC or Qatar or United Arab Emirates) (Topic) OR (("Health Insurance") or ("insured") Or ("uninsured") Or ("community insurance") Or ("medical insurance") Or ("health coverage")) And (Saudi Arabia or Bahrain or Kuwait or GCC or Qatar or United Arab Emirates) (Title) OR (("Health Insurance") or ("insured") Or ("uninsured") Or ("community insurance") Or ("medical insurance") Or ("health coverage")) And (Saudi Arabia or Bahrain or Kuwait or GCC or Qatar or United Arab Emirates) (Abstract) and 2022 or 2021 or 2020 or 2019 or 2018 or 2017 or 2016 or 2015 or 2014 or 2013 or 2012 (Publication Years) | English,10 years | 01/01/2012 to 31/10/2022 | 113 |
| CINAHL | TI ( ((health Insurance or insured or uninsured or community insurance or medical insurance or health coverage)) AND ((Saudi Arabia* or kingdom of saudi arabia* or ksa* or UAE* or *GCC* or "gulf" or United Arab Emirates* or Kuwait* or Oman* or Qatar*)) ) OR AB ( ((health Insurance or insured or uninsured or community insurance or medical insurance or health coverage)) AND ((Saudi Arabia* or kingdom of saudi arabia* or ksa* or UAE* or *GCC* or "gulf" or United Arab Emirates* or Kuwait* or Oman* or Qatar*)) ) | English,10 years | 01/01/2012 to 31/10/2022 | 108 |
